# A multidisciplinary approach to triage patients with breast disease during the COVID‐19 pandemic: Experience from a tertiary care center in the developing world

**DOI:** 10.1002/cnr2.1309

**Published:** 2021-01-13

**Authors:** Abida K. Sattar, Hania Shahzad, Adnan Abdul Jabbar, Azmina T. ValiMohammed, Sadaf Khan, Yasmin Vellani, Romana Idrees, Nasir Ali, Imrana Masroor, Humera Saeed, Gulzar Lakhani, Nadia Ayoub, Atif Waqar, Muhammad Zia‐ul Islam, Salman Kirmani, Asad Latif, Syed Ather Enam

**Affiliations:** ^1^ Department of Surgery Aga Khan University Karachi Pakistan; ^2^ Aga Khan University Karachi Pakistan; ^3^ Department of Oncology Aga Khan University Karachi Pakistan; ^4^ Department of Nursing Services Aga Khan University Hospital Karachi Pakistan; ^5^ Department of Pathology Aga Khan University Karachi Pakistan; ^6^ Department of Radiation Oncology Aga Khan University Karachi Pakistan; ^7^ Department of Radiology Aga Khan University Karachi Pakistan; ^8^ Department of Psychiatry Aga Khan University Karachi Pakistan; ^9^ Department of Pediatrics and Child Health/Section of Medical Genetics Aga Khan University Karachi Pakistan; ^10^ Department of Anesthesiology Aga Khan University Karachi Pakistan

**Keywords:** breast cancer, clinical outcome, surgical oncology

## Abstract

**Background:**

The COVID‐19 pandemic has created a need to prioritize care because of limitation of resources. Owing to the heterogeneity and high prevalence of breast cancers, the need to prioritize care in this vulnerable population is essential. While various medical societies have published recommendations to manage breast disease during the COVID‐19 pandemic, most are focused on the Western world and do not necessarily address the challenges of a resource‐limited setting.

**Aim:**

In this article, we describe our institutional approach for prioritizing care for patients presenting with breast disease.

**Methods and results:**

The breast disease management guidelines were developed and approved with the expertise of the Multidisciplinary Breast Program Leadership Committee (BPLC) of the Aga Khan University, Karachi, Pakistan. These guidelines were inspired, adapted, and modified keeping in view the needs of our resource‐limited healthcare system. These recommendations are also congruent with the ethical guidelines developed by the Center of Biomedical Ethics and Culture (CBEC) at the Sindh Institute of Urology and Transplantation (SIUT), Karachi.

Our institutional recommendations outline a framework to triage patients based on the urgency of care, scheduling conflicts, and tumor board recommendations, optimizing healthcare workers' schedules, operating room reallocation, and protocols. We also describe the “Virtual Blended Clinics”, a resource‐friendly means of conducting virtual clinics and a comprehensive plan for transitioning back into the post‐COVID routine.

**Conclusion:**

Our institutional experience may be considered as a guide during the COVID‐19 pandemic, particularly for triaging care in a resource‐limited setting; however, these are not meant to be universally applicable, and individual cases must be tailored based on physicians' clinical judgment to provide the best quality care.

## 1 INTRODUCTION

The COVID‐19 pandemic has challenged the world with unprecedented health, economic, and social consequences. According to the United Nations, it is the worst crisis that the Organization has faced in its 75‐year history.^1^ This pandemic has disrupted the delivery of healthcare services globally, posing additional risks to those requiring care unrelated to COVID‐19 owing to existing comorbidities. As of 13 May 2020, there have been more than 4 million confirmed cases of COVID‐19 and more than a quarter million deaths worldwide due to the virus.^2^ While the COVID‐19 outbreak affects all segments of the population, it has disproportionately affected vulnerable populations such as the elderly, the immunocompromised, patients with disabilities or pre‐existing comorbidities, and those belonging to the lower socioeconomic status. South Asia and Africa, which comprise a significant number of lower and middle income countries (LMIC) have reported at least 100 000 and 50 000 confirmed cases, respectively.^2^ While these developing countries share a smaller proportion of the confirmed global disease burden, this does not necessarily translate into milder outcomes for these regions. The developing world remains particularly at high risk for adverse outcomes due to poor pre‐pandemic healthcare infrastructure, limited resources, and economic costs of lockdowns. These challenges will be accentuated as the number of infected patients requiring critical care rises with severe limitation of resources in these countries.

Immunocompromised patients and those with cancer have been shown to have a higher incidence of complications and mortality from SARS COV.^3^ Breast cancer is one of the most prevalent cancers in both the developed and developing world. As hospital resources and staff become limited during the COVID‐19 pandemic, it becomes critically important to triage patients timely and appropriately. With regard to patients with breast diseases, it is imperative to differentiate between patients who require urgent care and patients whose care can be deferred without leading to worse outcomes. This is important to mitigate the risk to these patients and to preserve precious hospital resources.

Pakistan reported its first case of COVID‐19 on 26 February 2020, which was treated at our institution.^4^ Since then, Pakistan has reported a minimum of 35 788 confirmed cases with at least 770 confirmed deaths.^5^ The case fatality rate in Pakistan is 2.1% and the recovery rate is 28%.^6^ Pakistan is among the LMICs that do not have a strong healthcare infrastructure. In light of the pandemic, our institution aimed to implement strategies to effectively conserve resources and limit exposure of its healthcare staff while continuing to provide essential healthcare services to the patients. In this article, we aim to describe and share our “institutional guidelines” adopted to triage patients who require care for breast‐related issues, in our setting as an LMIC. There is often a gap between consensus recommendations by professional societies and their translation into practice, particularly in developing countries. Work like ours can help provide a template for utilization in other resource‐limited countries/hospitals/regions with modifications, as appropriate.^7^, ^8^, ^9^ These guidelines are meant to aid treatment decisions in these unprecedented circumstances of the COVID‐19 pandemic and emphasize multidisciplinary care and teamwork. They are not intended to supersede physician judgment or tumor board decisions and are subject to modification keeping in view available resources and COVID‐19 prevalence.

## 2 METHODS (DEVELOPMENT OF RECOMMENDATIONS)

The breast disease management guidelines were developed and approved by the multidisciplinary Breast Program Leadership Committee (BPLC) of the Aga Khan University, Karachi, Pakistan. These guidelines were inspired by COVID resources from the Society of Surgical Oncology (SSO) and American Society of Breast Surgeons (ASBrS) and subsequently adapted and modified keeping in view the needs of our resource‐limited healthcare systems. They may be utilized for other resource‐limited countries/hospitals/regions with modifications, as appropriate.^7^, ^8^, ^9^ These recommendations are also congruent with the ethical guidelines developed by the Center of Biomedical Ethics and Culture (CBEC) at Sindh Institute of Urology and Transplantation (SIUT), Karachi.^10^

## 3 RESULTS

### 3.1 Triaging patients based on breast pathology

Patients are triaged based on existing pathology, tumor type, available treatment options, and the urgency of the need for surgical treatment. Surgical procedures put the patients as well as healthcare providers at a heightened risk of exposure to SARS COV and have the potential to deplete medical supplies and occupy beds that are needed for the COVID‐19 patients. Therefore, a patient's pathology is the primary focus, with the intent of deferring surgery where safely possible, without adversely impacting the quality of care and prognosis.^11^

**FIGURE 1:**
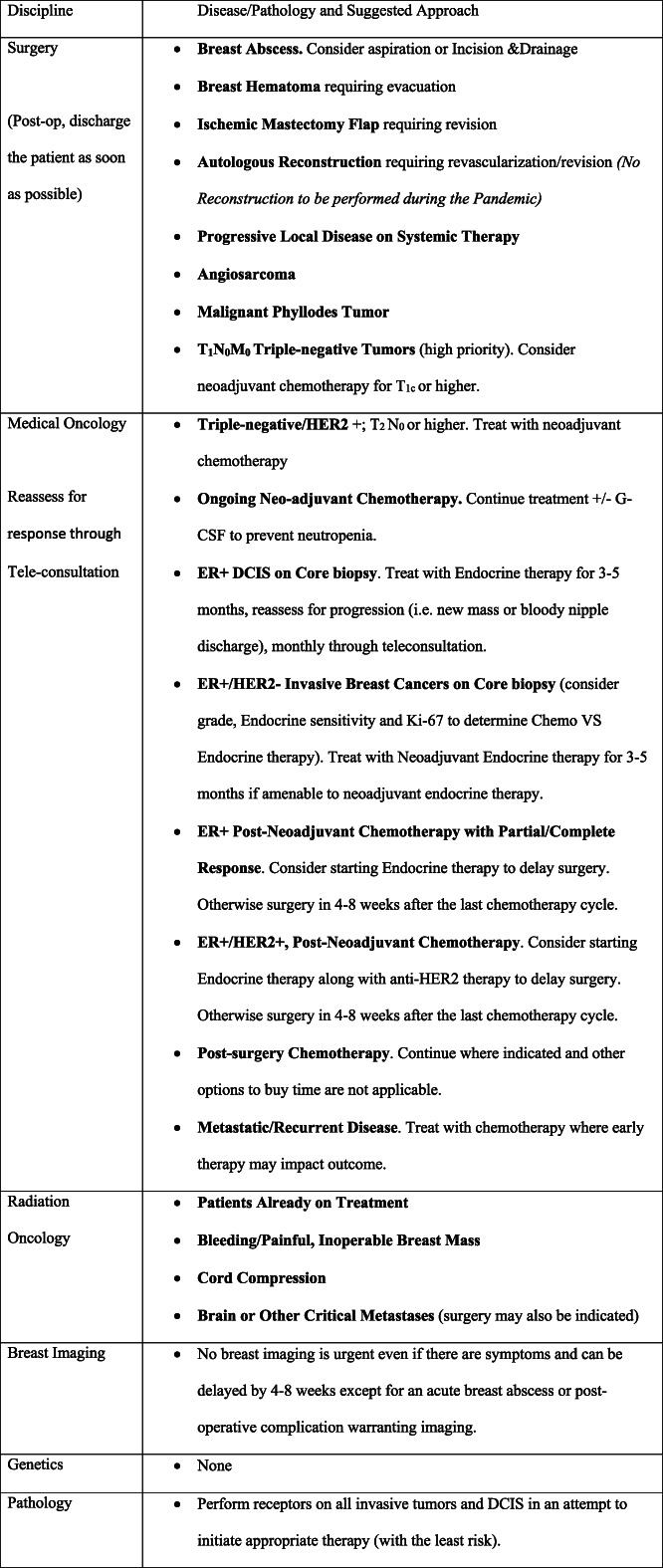
Priority A: Urgent Care

**FIGURE 2:**
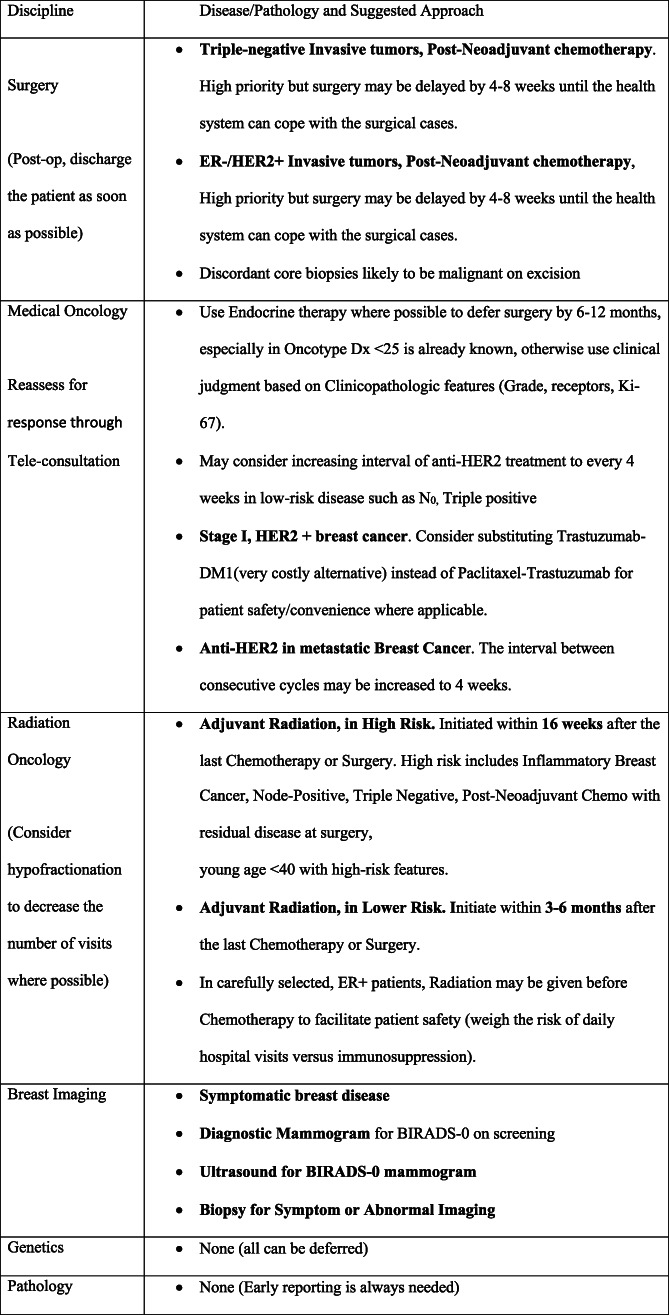
Priority B: Intervention may be altered or delayed for 4–8 weeks

**FIGURE 3:**
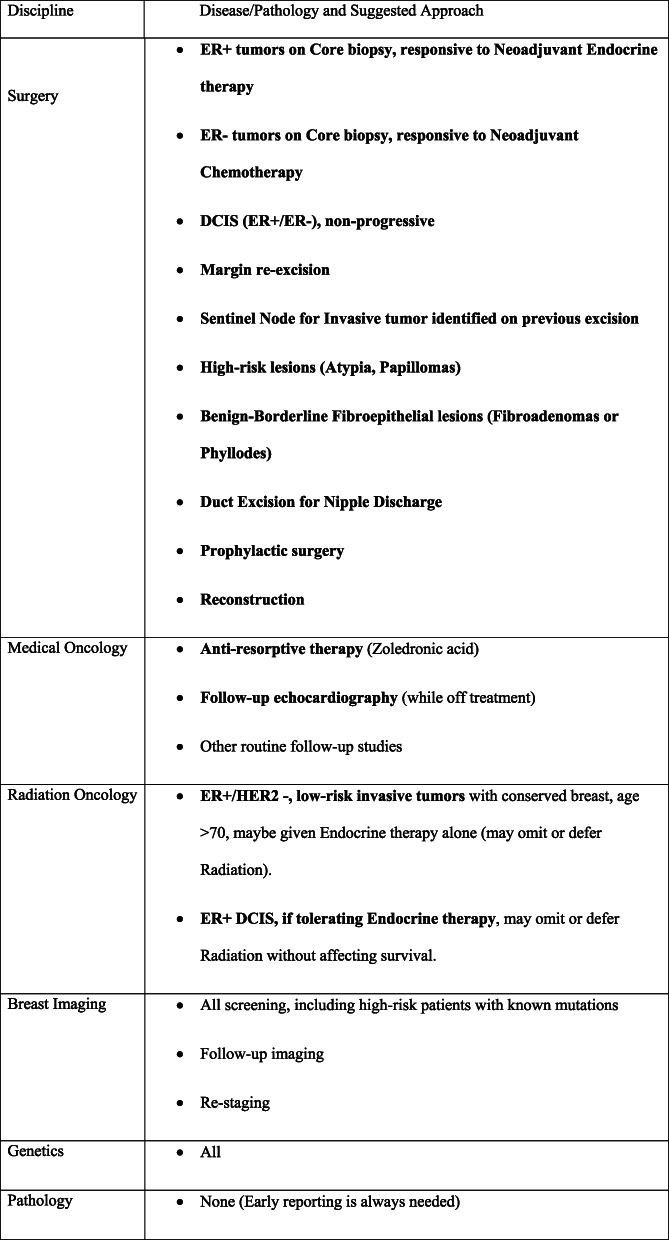
Priority C: Intervention may be delayed until the pandemic is under control*. *Assuming the pandemic will be under control in 3–4 months

- Priority A includes all those patients who have conditions that are immediately life‐threatening or symptomatic requiring urgent treatment (see Figure 1).
- Priority B includes all those patients who have conditions that do not require immediate treatment but should start treatment before the pandemic is under control. Intervention in priority B may be altered or delayed for 4–8 weeks (see Figure 2).
- Priority C includes all those patients who have conditions that can be safely deferred until after the pandemic is under control. These groupings were defined for each specialty such as surgery, medical oncology, radiation oncology, breast imaging, genetics, and pathology to facilitate decision making in the respective disciplines (see Figure 3).

**TABLE 1:** Operating room reallocation: Column: Operating room number; Row: Day of the week; Yellow: Operating rooms not in use; Red: Operating room for urgent/emergent care; Pink: Breast Surgery/General Surgery; Blue: Operating room reserved for Suspected/Confirmed COVID cases

Day	OR 1	OR 2	OR 3	OR 4	OR 5	OR 6‐7	OR 8	OR 9	OR 10	OR 11	OR 12	OR 13	OR 14	OR 15	OR 16	OR 17
**MON**	**Gynae**	**BS/GS**	**Closed**	**ENT**	**URGENT/EMERGENT**	**CLOSED**	**URGENT/EMERGENT**	**Cardiac (adults)**	**Cardiac (peds)**	**Neuro**	**Ortho**	**CLOSED**	**Optho**	**SUSPECTED OR CONFIRMED COVID‐19 CASES ONLY**		
**TUES**	**Gynae**	**Ped**	**BS/GS**	**Uro**				**Cardiac (adults)**	**Cardiac (peds)**	**Neuro**	**Ortho**		**Optho**			
**WED**	**BS/GS**	**Ped**	**BS/GS**	**Vasc**				**Cardiac (adults)**	**Cardiac (peds)**	**Neuro**	**Ortho**		**CLOSED**			
**THURS**	**Gynae**	**Plast**	**BS/GS**	**ENT**				**Cardiac (adults)**	**CLOSED**	**Neuro**	**Ortho**					
**FRI**	**Vasc/Plast**	**Uro**	**BS/GS**	**ENT**				**Cardiac (adults)**		**Neuro**	**Ortho**		**Pain/Dental**			

*Note*: Key: Gynaecology (Gynae), Breast surgery (BS), General surgery (GS), Plastic surgery (Plast), Vascular surgery (Vasc), Pediatric surgery (Peds), Urology (Uro), Otolaryngology (ENT), Cardiothoracic surgery (Cardiac adults), Pediatric cardiac surgery (Cardiac Peds), Neurosurgery (Neuro), Orthopedics (Ortho), Ophthalmology (Optho), Pain procedures (Pain), Dental/oral Surgery (Dental).

### 3.2 Tumor board

A multidisciplinary tumor board meets through video conference once a week where only cases warranting decision making are discussed. For an informed decision, history, physical exam findings, other comorbidities, relevant images, and pathology are reviewed, and treatment decisions are made by placing each patient in one of the priority groups A, B, or C. Where definitive care is deferred, 4–6 weeks follow‐ups are planned to monitor the patients. Based on these follow‐ups and changes in a patient's status, the categorization may change.

### 3.3 Healthcare workers' schedule

To minimize exposure to healthcare professionals (HCPs), the daily schedule is planned on a rotational basis for all providers including nurses and technicians, allowing each person at least 1 to 2 days off in a week and, where possible, up to seven consecutive days. In certain specialties, in‐patient team schedules have been formulated to accommodate a week on and a week off schedule for healthcare providers to minimize exposure. A third team is kept as a backup to replace any staff that warrants isolation/quarantine.

### 3.4 Operating rooms reallocation

Operating rooms (OR) were reallocated to preserve personal protective equipment supplies and limit the exposure to OR personnel by maintaining a rotational schedule. From our usual process of assigning ORs to a particular surgeon, we switched to assigning operating rooms based on specialty. An OR suite consisting of three ORs, a pre‐op area, and a recovery room that were independent of the other 14 ORs were converted into negative pressure rooms and reserved for COVID‐suspected or COVID‐positive patients only. All semi‐elective surgical cases undergo a COVID test 48 hours before surgery. For cost containment, the test is performed as a pooled test of eight patients using PCR. If the pool tests positive, each sample is re‐run individually. With the expected rate of positive samples being under 10%, this has been cost‐effective (Table 1).

### 3.5 Operating room protocol

**FIGURE 4:**
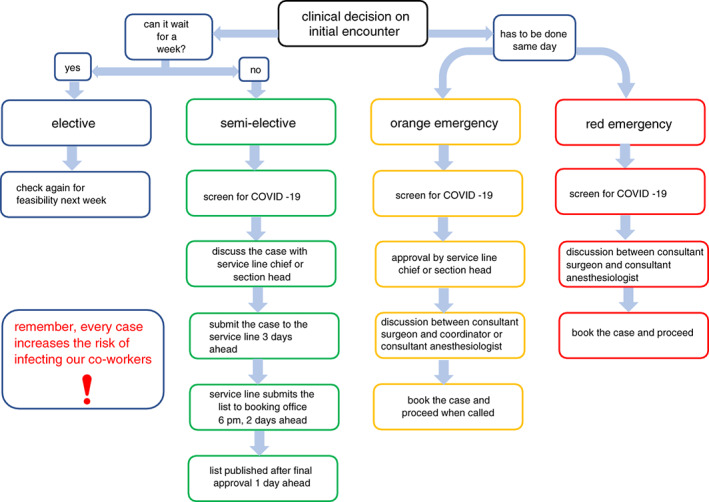
Process flow for surgical procedures^12^

Since all specialties use the OR)facilities, a color‐coded system is in place to prioritize cases based on urgency. Refer to the OR flowchart (see Figure 4). The following color codes are followed in the flow chart.

- **Red emergency**: Urgent/emergent/life or organ‐threatening surgery to be performed within 1 h. If the emergency OR is occupied, any nonurgent case is delayed/procedure will be stopped, preferably in the same specialty where possible.
- **Orange emergency or Semi‐emergency**: Such cases are taken up as soon as OR becomes available or semi‐elective list is completed for the day for that particular specialty.
- **Semi‐Elective**: All breast oncology cases are in this category.

### 3.6 Approval of cases by clinical leaders

To maintain transparency and adherence to all institutional guidelines, all surgical cases need to be approved by the Section Heads/Division Chiefs/Chairs of the Department of Surgery and Anesthesia before being confirmed on the surgical schedule. The approvals are in line with the institutional guidelines, Figures 1, 2, 3. From the period 18 March through 12 May 2020, a decrease in surgical volume was seen when compared to the same period in 2019 (48 breast surgical procedures in 2019 vs 30 breast surgical cases in 2020).

### 3.7 Virtual and blended clinics

New patients with urgent issues such as lumps or known cancers are given urgent appointments. Nonurgent issues are deferred where possible or addressed via virtual clinics. New patients are seen in “Blended Clinics.” In a blended clinic, a patient arrives at the hospital/clinic building and is met by a screening team in the open, where a brief, COVID‐focused history and temperature are taken. If the patient is considered high‐risk, he or she is referred to the designated COVID testing area. If cleared for a physical clinical encounter, the patient is allowed to proceed into the clinical area and get registered. In‐person visits are spaced out on the schedule to prevent crowding of the clinical areas. Seating in the waiting area has been rearranged to maintain physical distance. All patients are also mandated to wear a mask and may be accompanied by only one attendant. In a blended clinic, the patient encounter is initiated with a phone call from the physician to the patient in the waiting room. A detailed history is taken and images are reviewed. The patient is then invited to the clinical exam area for a brief face‐to‐face encounter with the clinician, who uses personal protective equipment (PPE). This is followed up with a second phone call for counseling and instructions. Using this technique, we have been able to limit exposure times without compromising care.

Most clinic slots are utilized for follow‐up patients with unresolved issues such as ongoing investigations for the diagnosis of malignancy or those on active treatment. All follow‐up appointments are virtual. Since many of our patients come from less developed parts of the country, they may not have access to a computer/internet or may not have the technical expertise to use these services. Therefore, our virtual clinics are based on telephonic conversations. If there is a need for a physical exam, and the patient is in close proximity to the hospital, they are seen the same day or during the next clinic. Patients may elect to send their images of a wound or a lesion as necessary over a freeware, cross‐platform messaging, and voice over IP service called WhatsApp (popular in Pakistan) to the “Breast Hotline” number. The hotline phone is carried by our senior breast surgery nurse 24 hours a day. Prescriptions and requests/orders for imaging as well as details of follow‐ups are also sent to the patients through WhatsApp. All routine survivor visits and routine follow‐up imaging have been deferred by 3 months.

### 3.8 Prescriptions

Patients have the option of filling in their prescriptions virtually by sending a copy of the prescription though email or WhatsApp to the AKU Pharmacy. The dispensed medication is then delivered to the patient's home, thus obviating the need for the patient to leave home.

### 3.9 Transition plan

Now that we have been successfully following the above plan for over a month, we are now in the process of developing a strategy to smoothly transition back into usual routine care. The transition will be slow and deliberate. Lists of patients that were triaged into groups B and C are maintained. When the COVID curve starts to flatten, we will be able to contact our patients from groups B and C and prioritize their surgical care as needed along with those who may need urgent care. How long the tele‐clinics will continue will be partly dictated by the COVID disease burden and hesitancy of patients to attend in person. The silver lining may be the opportunity to experiment with this model and determine its success, especially in patients who come from far‐flung areas to our academic medical center to seek specialized care. In the long term, allocating a certain number of clinic slots for teleconsultation may be an opportunity to offer expert advice to patients in remote areas or even patients outside the country.

## 4 DISCUSSION

COVID‐19 pandemic has disrupted the global healthcare infrastructure. While cancer management is already challenging in resource‐limited settings, recommendations from the international guidelines are applicable to low resource settings in many cases. For example, wearing cloth masks in public and saving surgical masks to be used within the health facilities is considered convenient to LMIC. Also, educational materials and programs directed to cancer patients and promoting proper hygiene and infection prevention measures are feasible.^13^

However, these recommendations may be limited by complex geographic situations, unavailability of equipment or means of communication (telemedicine), limited expertise of the staff, and local needs of individual countries.^14^ The uncertainty of the pandemic's duration, the strain on resources during the pandemic, and the impact of BC treatment delay on survival, along with the known BC situation in LMIC, mandate the implementation of resource‐stratified guidelines as previously suggested by Breast Global Health Initiative.^15^

Consequently, several guidelines have been published to manage cancer care in resource‐limited settings and with a consensus to triage and prioritize cancer surgery aimed to preserve the patient's function, long‐term prognosis, and quality of life. The focus of current cancer surgery care has shifted into the establishment of definitive diagnosis, staging, and selected essential surgery. These guidelines explore the possibility of application in LMICs and hypothesize their practicality. In contrast, we describe our experience, which has been in place since the beginning of the COVID‐19 pandemic. With adaptions and alterations to suit the need of the hour, our guidelines provide a framework for countries that may be struggling to manage a high influx of COVID‐19 cases.

Furthermore, published literature for breast disease management in LMICs details the criteria of triaging patients for screening, imaging, work‐up, surgical, medical, and radiation oncology, follow‐up care, post‐operative management, supportive care, and palliation. Apart from detailing the implemented and workable guidelines of the above‐mentioned categories, our recommendations also describe prioritization in genetics, pathology, staff management, tumor board conductance, operating room protocols, resource allocation, as well as a transition‐back plan. The above‐mentioned categories together form a holistic multidisciplinary approach and can also be implemented in settings where an infrastructure was previously not in place and requires a new pandemic set‐up.

For settings that lack a high‐quality telecommunication network to conduct long‐distance telecommunication, we provide a cost‐effective concept of “blended” clinics. Blended clinics can minimize the patient/physician contact without compromising the quality of care, whose implementation requires a basic infrastructure consisting of a low‐quality internet/landline network and availability of PPE.

In a nutshell, almost all recommendations describe means of delaying less urgent treatment and prioritizing breast cancer care carefully based on individual patients' needs as dictated by the disease, ground reality, and resources.^13^, ^14^, ^16^

## 5 CONCLUSION

In the wake of the COVID‐19 pandemic, as hospital resources and staff become limited, while it is important to prioritize care, it is equally important for the HCPs and the institutional leadership to communicate clearly and regularly. No plan can be etched in stone and all stakeholders have to be ready and willing to respond quickly to the changing needs based on rapidly changing circumstances. Owing to a lack of existing evidence, our experience may be considered as a guide during the COVID‐19 pandemic, particularly for triaging care in a resource‐limited setting.

## CONFLICT OF INTEREST

All authors report no conflict of interest relevant to this article.

## AUTHOR CONTRIBUTIONS

*Conceptualization*, A.K.S., H.S., A.J., A.V.M., S.K., Y.V., R.I., N.A., I.M., H.S., G.L., N.A., A.W., M.I., S.K., A.L., S.A.E..; *Methodology*, A.K.S., H.S.; *Project Administration*, A.K.S.; *Resources*, A.K.S.; *Supervision*, A.K.S.; *Validation*, A.K.S.; *Visualization*, A.K.S.; *Writing Original Draft*, A.K.S., H.S.; *Writing Review Editing*, A.K.S., H.S., A.J., A.V.M., S.K., Y.V., R.I., N.A., I.M., H.S., G.L., N.A., A.W., M.I., S.K., A.L., S.A.E.

## ETHICAL STATEMENT

Ethical approval was not required as no patient‐related data was utilized in writing this article. The article reflects the experience of the authors.

## Data Availability

Data sharing does not apply to this article as no new data were created or analyzed in this study.
